# Association of Apolipoprotein A5 Gene Variants with Hyperlipidemic Acute Pancreatitis in Southeastern China

**DOI:** 10.1089/gtmb.2023.0107

**Published:** 2023-09-27

**Authors:** Yingyi Li, Hehui Cai, Yancheng Lin, Zhipeng Huang, Apei Zhou, Tianhao Huang, Yue-e Zeng, Meizhen Ye, Guiyuan Guo, Zicheng Huang

**Affiliations:** ^1^ Department of Gastroenterology, The First Hospital of Quanzhou Affiliated to Fujian Medical University, Quanzhou, China.; ^2^ Clinical Laboratory, The First Hospital of Quanzhou Affiliated to Fujian Medical University, Quanzhou, China.; ^3^ HI. Q Biomedical Laboratory, Taiwan Investment Zone, Quanzhou, China.

**Keywords:** apolipoprotein A5, rs651821, hyperlipidemic acute pancreatitis, serum triglyceride

## Abstract

**Background:**

Apolipoprotein A5 (APOA5) is involved in serum triglyceride (TG) regulation. Several studies have reported that the rs651821 locus in the *APOA5* gene is associated with serum TG levels in the Chinese population. However, no research has been performed regarding the association between the variants of rs651821 and the risk of hyperlipidemic acute pancreatitis (HLAP).

**Methods:**

A case-control study was conducted and is reported following the STROBE guidelines. We enrolled a total of 88 participants in this study (60 HLAP patients and 28 controls). *APOA5* was genotyped using PCR and Sanger sequencing. Logistic regression models were conducted to calculate odds ratios and a 95% confidence interval.

**Results:**

The genotype distribution of the rs651821 alleles in both groups follow the Hardy-Weinberg distribution. The frequency of the “C” allele in rs651821 was increased in HLAP patients compared to controls. In the recessive model, subjects with the “CC” genotype had an 8.217-fold higher risk for HLAP (OR = 8.217, 95% CI: 1.023–66.01, *p* = 0.046) than subjects with the “TC+TT” genotypes. After adjusting for sex, the association remained significant (OR = 9.898, 95% CI: 1.176–83.344, *p* = 0.035). Additionally, the “CC” genotype was related to an increased TG/apolipoprotein B (APOB) ratio and fasting plasma glucose (FPG) levels.

**Conclusions:**

Our findings suggest that the C allele of rs651821 in *APOA5* increases the risk of HLAP in persons from Southeastern China.

## Introduction

The prevalence of hypertriglyceridemia (HTG) has continued to increase rapidly over recent years in China (Opoku et al., 2019; Pan et al., 2016; Zhang et al., 2017). A cross-sectional study suggested that the prevalence of HTG has reached 43.9% in northeast China (Zhang et al., 2017). HTG is a lipid metabolism disorder associated with an increased risk of atherosclerotic cardiovascular disease and all-cause mortality (Nordestgaard and Varbo, 2014). In addition, severe HTG (SHTG triglyceride [TG] ≥5.65 mmol/L) can cause hyperlipidemic acute pancreatitis (HLAP). Moreover, a higher TG level is associated with a higher risk of acute pancreatitis (AP) (Hernandez et al., 2021). About 20% of individuals with serum TG levels >11.3 mmol/L could develop AP (Hansen et al., 2021).

Recent studies suggested that HTG had become the second leading cause of AP in several regions of China, accounting for ∼10–30% of AP cases (Boxhoorn et al., 2020; Chen et al., 2019; Du et al., 2019; He and Lyu, 2018; Lee and Papachristou, 2019; Zhang et al., 2019; Zheng et al., 2020). There are some evidences that HLAP may be prone to aggravation and recurrence, and have a higher rate of complications compared with other causes of pancreatitis, such as pulmonary infection, circulatory and respiratory failure, and renal insufficiency (Guo et al., 2017; He and Lyu, 2018; Yang et al., 2017). It could severely affect the patient's wellness and thus consume more medical resources in the next decade.

The pathogenesis of HLAP is not yet clear. Free fatty acids, calcium overload, endoplasmic reticulum stress, microcirculation disorders, oxidative stress, inflammatory mediators and cytokine damage, and gene polymorphisms are all involved in the occurrence and development of HLAP (Guo et al., 2019). Among them, the genetic predisposition plays a critical role. Particularly, variants in the lipoprotein lipase (*LPL*) gene and its molecular regulating genes could cause chylomicronemia and recurrent pancreatitis (Goldberg and Chait, 2020; Paquette et al., 2021).

Apolipoprotein A5 (*APOA5*) gene is a member of the *APOA1/APOC3/APOA4/APOA5* gene cluster, which is located on chromosome 11q23. It encodes an apolipoprotein involved in regulating the serum TG and high-density lipoprotein cholesterol (HDL-C) concentrations (Pennacchio et al., 2001). Several single nucleotide polymorphisms (SNPs) of the *APOA5* gene have been reported to affect the plasma TG levels in different populations and are significantly related to many diseases such as obesity, hypertension, coronary heart disease, metabolic syndrome, and stroke (Fu et al., 2015; Gao et al., 2018; Gombojav et al., 2016; Kefi et al., 2017; Ken-Dror et al., 2010; Kim et al., 2019; Lim et al., 2017; Saleheen et al., 2010; Wang et al., 2020; Wu et al., 2016; Xiao et al., 2017; You et al., 2018; Zhu et al., 2014).

Genome-wide association studies (GWAS) in the Chinese population identified an SNP (rs651821) in *APOA5* that was significantly associated with serum TG level (Tan et al., 2012; Zhu et al., 2017). However, the association between the rs651821 in the *APOA5* gene and HLAP remains unknown so far. This study aims to investigate the relationship between the *APOA5* rs651821 and HLAP in the Chinese population. The following article was presented in accordance with the STROBE reporting checklist.

## Methods

### Study design

A case–control study was conducted following Strengthening the Reporting of Observational Studies in Epidemiology (STROBE) statements. The cases were represented by patients diagnosed with HLAP in the hospital, the controls were patients with biliary acute pancreatitis (BAP). The sample was obtained from the Fujian Medical University Affiliated First Quanzhou Hospital, located in southeastern China, during 2018–2022. This study was approved by the Ethics Committee of the Fujian Medical University Affiliated First Quanzhou Hospital.

### Subjects

A total of 88 participants were recruited, including 60 HLAP patients as cases and 28 BAP patients as controls. The inclusion criteria for the case group were (1) diagnosis of AP according to Chinese guidelines for the management of AP (at least two of the following three features: upper abdominal pain consistent with AP, serum lipase activity exceeds the upper limit of normal by three times, radiographic features of AP); (2) confirmed dyslipidemia with serum TG >11.3 mmol/L or serum TG >5.65 mmol/L together with lipemia. The inclusion criteria for the control group were (1) diagnosis of AP according to Chinese guidelines for the management of AP; (2) confirmed gallstones or biliary sludge on imaging methods or elevated serum levels of alanine aminotransferase (>60 U/L).

Patients with a history of alcohol consumption of >20 g/day, a history of pancreatic toxic drugs, known pancreatic or periampullary tumors, pancreatic duct anomaly disorders, hereditary pancreatitis, autoimmune pancreatitis, and traumatic pancreatitis were excluded from the study. All patients were not genetically related and were grouped by clinical presentation alone. The participants did not know their genotypes in advance, and neither the researchers.

### Biochemical measurements

The general information, such as age, gender, alcohol consumption, and smoking status was collected from medical history and questionnaires. Blood samples were obtained in the morning after an overnight fast for detecting biochemical parameters, including lipid parameters, glycemia parameters, and liver function.

### Genotyping analysis

Genomic DNA was extracted from peripheral blood lymphocytes. PCR reactions were performed on gDNA with the primer pairs (5′ AGCTACGGAGTTGTCAAGGC 3′ as the forward primer and 5′ GAACTGTTCCTGGGGTCTGG 3′ as the reverse primer). The PCR products of the expected size were separated by 1% agarose gel electrophoresis to confirm the presence and specificity of targeted sequences. After further purification, the PCR products were sequenced by the dideoxy approach. DNA Baser v5.15.0 was used to align the DNA fragments to target references and detect the variants from chromatograms.

### Statistical analysis

Quantitative variables were expressed as mean ± standard deviation. The Hardy–Weinberg equilibrium was assessed with the chi-square test (*p*-value >0.05). Differences in the characteristics between groups were analyzed using the Student's *t*-test or Wilcoxon test. Differences in genotype distribution and allele frequency between groups were compared with the chi-square test or Fisher's exact test. Statistical analysis was performed using SPSS 22.0 software, and the *p*-value <0.05 was considered statistically significant.

## Results

### General characteristics

The general characteristics of the 60 patients with HLAP and the 28 controls are presented in Table 1. All the participants are Chinese. The HLAP group was younger and had more male patients than the BAP group. The mean total cholesterol (TC), TG, non-high-density lipoprotein cholesterol (non-HDL-C), and glycated hemoglobin (HbA1c) were significantly higher in the HLAP group, but the systolic blood pressure (SBP), low-density lipoprotein cholesterol (LDL-C) was significantly higher in BAP group. The two groups did not differ by diastolic blood pressure (DBP), high-density lipoprotein cholesterol (HDL-C), or fasting plasma glucose (FPG) levels. The proportion of smokers, drinkers, and patients with fatty liver and diabetes was higher in the HLPA group and the proportion of patients with hypertension was higher in the BAP group.

**Table 1.** Clinical and Biochemical Characteristics of Patients with Hyperlipidemic Acute Pancreatitis and Controls

	HLAP patients (*n* = 60)	BAP controls (*n* = 28)	*t *(*χ*^2^)	*p*
Male, *n* (%)	43 (71.7)	12 (42.9)	6.761	0.009^*^
Age (years)^a^	38.650 ± 9.016	58.790 ± 14.635	36.881	<0.001^*^
SBP (mm Hg)^a^	132.700 ± 15.854	144.464 ± 25.606	36.984	0.031^*^
DBP (mm Hg)^a^	84.917 ± 10.888	84.852 ± 17.033	37.653	0.987
TC (mmol/L)^a^	11.924 ± 4.627	4.653 ± 0.975	69.394	<0.001^*^
TG (mmol/L)^a^	29.025 ± 18.777	1.256 ± 0.584	59.245	<0.001^*^
HDL-C (mmol/L)^a^	0.949 ± 0.840	1.073 ± 0.284	77.960	0.318
LDL-C (mmol/L)^a^	2.029 ± 1.773	3.025 ± 0.720	82.309	<0.001^*^
Non-HDL-C (mmol/L)^a^	11.018 ± 4.356	3.580 ± 0.895	66.333	<0.001^*^
FPG (mmol/L)^a^	12.507 ± 6.038	10.134 ± 4.884	86.000	0.072
HbA1c (%)	8.537 ± 2.747	6.349 ± 1.238	82.921	<0.001^*^
Drinking, *n* (%)	23 (38.3)	1 (3.6)	9.944	0.002^*^
Smoking, *n* (%)	24 (40.0)	2 (7.1)	9.901	0.002^*^
Hypertension, *n* (%)	11 (18.3)	11 (39.3)	4.470	0.034^*^
Fatty liver, *n* (%)	52 (86.7)	11 (39.3)	21.073	<0.001^*^
Diabetes, *n* (%)	35 (58.3)	8 (28.6)	6.768	0.009^*^

^a^ Results are expressed as mean ± SD.

^*^ *p* < 0.05 was considered statistically significant.

BAP, biliary acute pancreatitis; DBP, diastolic blood pressure; FPG, fasting plasma glucose; HbA1c, glycated hemoglobin; HDL-C, high-density lipoprotein cholesterol; HLAP, hyperlipidemic acute pancreatitis; LDL-C, low-density lipoprotein cholesterol; SBP, systolic blood pressure; SD, standard deviation; TC, total cholesterol; TG, triglyceride.

### Genotype distribution comparison

The distribution of rs651821 in *APOA5* was in accordance with the Hardy–Weinberg distribution in all participants as well as separately in HLAP patients and controls (*p* > 0.05). The genotype distributions and allele frequencies of rs651821 is shown in Table 2. The frequency of the “TT,” “TC,” and “CC” genotypes was 38.3%, 38.3%, and 23.3% in the HLAP patients, and 53.6%, 42.9%, and 3.6% in BAP controls. Meanwhile, a significant elevation was observed in the frequency of the “C” allele in rs651821 for the HLAP patients (42.5% vs. 25.0% for BAP controls, *p* = 0.025) in Table 2.

**Table 2.** The Genotype and Allele Distributions of rs651821 in Hyperlipidemic Acute Pancreatitis Patients and Controls

SNP	Genotype/allele		HLAP patients (*n* = 60),* n *(%)	BAP controls (*n* = 28),* n *(%)	*χ* ^2^	*p*
rs651821	Genotype	TT	23 (38.3)	15 (53.6)	5.499	0.064
		CT	23 (38.3)	12 (42.9)		
		CC	14 (23.3)	1 (3.6)		
	Allele	T	69 (57.5)	42 (75.0)	5.020	0.025^*^
		C	51 (42.5)	14 (25.0)		

^*^ *p*-Value <0.05 was considered statistically significant.

SNP, single nucleotide polymorphism.

### APOA5 rs651821 and the risk of HLAP

Table 3 shows the relationship between *APOA5* rs651821 and the risk of HLAP. In the recessive model, we observed a significant association between HLAP risk and rs651821. Subjects with the “CC” genotype had an 8.217-fold higher risk for HLAP (odds ratio [OR] = 8.217, 95% confidence interval [CI]: 1.023–66.01, *p* = 0.046) than subjects with the “TC + TT” genotype. The association remained significant after controlling for sex (adjusted OR = 9.898, 95% CI: 1.176–83.344, *p* = 0.035).

**Table 3.** Apolipoprotein A5 rs651821 and the Risk of Hyperlipidemic Acute Pancreatitis with Recessive Model

Genotype	CC	CT+TT
HLAP patients (*n* = 60), *n* (%)	14 (23.3)	46 (76.7)
BAP controls (*n* = 28), *n* (%)	1 (3.6)	27 (96.4)
OR (95% CI)	1.023–66.017	1
*p*	0.046^*^	
Adjusted OR (95% CI)^a^	1.176–83.344	1
*p* ^*^	0.035^*^	

^a^ Adjusted for sex; ^*^p-value <0.05 was considered statistically significant.

OR, odds ratio.

### Association between genotypes and general characteristics

The differences in lipid parameters and glycemia parameters between the “CC” and the “TT” genotypes are presented in Table 4. Compared with the “TT” genotype, subjects with the genotype “CC” had significantly increased FPG and TG/apolipoprotein B (APOB) levels (*p* < 0.05).

**Table 4.** Differences of Parameters Between Genotypes (CC, TT)

rs651821	Genotype		*p*
	CC	TT	
Age (year)	38.400 ± 9.679	45.320 ± 15.968	0.192
TC (mmol/L)	10.807 ± 4.069	9.123 ± 5.010	0.110
TG (mmol/L)	27.643 ± 19.026	19.326 ± 22.739	0.086
HDL-C (mmol/L)	1.058 ± 0.882	0.993 ± 0.647	0.518
LDL-C (mmol/L)	1.697 ± 1.474	2.599 ± 1.805	0.179
Non-HDL-C (mmol/L)	9.749 ± 3.546	8.022 ± 5.020	0.104
TG/APOB	361.102 ± 1022.564	63.191 ± 122.000	0.017^*^
FPG (mmol/L)	13.509 ± 4.841	10.891 ± 6.680	0.043^*^
HbA1c (%)	7.979 ± 2.149	8.858 ± 2.934	0.428

^*^ *p*-value <0.05 was considered statistically significant.

APOB, apolipoprotein B; HbA1c, glycated hemoglobin; Non-HDL-C, non-high-density lipoprotein cholesterol.

## Discussion

Despite the rising incidence and the increased disease burden of HLAP, the genetic predisposition of it is always under-recognized (Baass et al., 2020). In this study, we evaluated the association between rs651821 in *APOA5* and HLAP risk in the southeast of China. We found that the minor allele “C” of rs651821 was associated with an increased HLAP risk. The “CC” genotype had a higher level of TG/APOB and FPG compared with the “TT” genotype.

The etiology of HLAP is complex and may involve multiple gene–gene and gene–environment interactions. APOA5 is essential LPL activator, which is involved in regulating TG metabolism (Fu et al., 2015). Its dysfunction may impact LPL activity and availability, and this then results in dyslipidemia (Kersten, 2014). Several SNPs of the *APOA5* gene were identified to be associated with increased TG levels in China, including rs651821 (Fu et al., 2015; Gombojav et al., 2016; Tan et al., 2012; You et al., 2018; Zhu et al., 2017).

*APOA5* variants were also found to be the most frequent genetic determinant of multifactorial chylomicronemia syndrome, and the second most frequent genetic determinant of familial chylomicronemia syndrome next only to *LPL*, which are characterized as recurring HLAP (D'Erasmo et al., 2019; Dron et al., 2020). Both rare and common variants that affect the concentration and activity of APOA5 may be one of the causative factors of HLAP.

Several SNPs in *APOA5* have been detected in some HLAP case reports (Chokshi et al., 2014; Hooper et al., 2014; Koopal et al., 2019; Perrone et al., 2019). Pu et al. (2020) demonstrated a robust association of rs2075291 in *APOA5* with HLAP, particularly in pregnancy. But data on the possible relation between HLAP and rs651821 in *APOA5* have so far been sparse. The SNP rs651821 was located in the 5′UTR and promoter region of *APOA5*. The frequency of variant rs651821 is 29% in the East Asian population, more prevalent than other populations in the world (Chou et al., 2022).

Chou et al. (2022) revealed variant rs651821 is the causal variant affecting TG level rather than variant rs662799 in the genome-wide association study (GWAS) of hyperlipidemia. A cross-sectional and representative survey of Jilin Province, China showed that the “C” allele frequency was 36.8%, and the frequency of the “TT,” “TC,” and “CC” genotypes was 39.8%, 46.7%, and 13.5% in the HTG group. Another prospective cohort study from Denmark showed that more AP events occurred in variant rs651821 carriers, but the frequency of variant rs651821 is low in the Denmark population (Hansen et al., 2021).

Our available data showed that the “C” allele frequency was 42.5%, and the frequency of the “TT,” “TC,” and “CC” genotypes was 38.3%, 38.3%, and 23.3% in the HLAP group. Moreover, we found that the genotypic and allelic frequencies of rs651821 exhibited significant differences between the HLAP and the BAP groups. We used the recessive genetic model, which made biological sense based on previous literature to establish an association between the genotype and HLAP risk (Adams et al., 2014; Lim et al., 2017). Furthermore, we found the carriers of the “CC” genotype of rs651821 were found to have an 8.217-fold higher risk of HLAP than those with other genotypes, which suggested that *APOA5* variant rs651821 conferred susceptibility to HLAP in the Quanzhou population.

Mechanistically, it has been proposed that chylomicrons (CM) excess is needed to provoke HLAP (Hernandez et al., 2021). Gonzales et al. demonstrated that the calculation of serum TG/APOB ratio may help identify HTG patients at risk for HLAP. This marker of CM persistence (TG/APOB >10.6) has a high sensitivity (90%) and specificity (75%) for the identification of HLAP risk (Bai et al., 2019). In our study, subjects with the genotype “CC” had a higher level of TG/APOB.

It showed that those with the genotype “CC” of rs651821 had a higher risk for HLAP. Our study also found that subjects with the genotype “CC” had a higher level of FPG. Previous research suggested that the genotype “AA” of rs1263173, which is also located in the 5′UTR region of *APOA5*, significantly increased the plasma glucose level (Xu et al., 2018). The rs1263173 “AA” carriers and rs651821 “CC” carriers may be more susceptible to type 2 diabetes mellitus. Accordingly, further research on the relationship between *APOA5* variant rs651821 and plasma glucose regulation is needed.

There are some limitations of our study that should be acknowledged. First, the sample size of our study cohort was small. Hence, the subjects in this study may not be entirely representative of the general southeastern Chinese population. Second, there was a difference in age between the two groups due to the features of the disease itself, so maybe some selection biases existed. Third, our study did not include other etiologies of AP such as alcoholic pancreatitis, autoimmune pancreatitis, and drug-induced pancreatitis.

We excluded the patients with alcoholic pancreatitis because excessive alcohol intake can lead to elevated TG and the disturbance of lipid metabolism, which could potentially introduce unnecessary confounding factors in the downstream analysis. Likewise, we excluded drug-induced and autoimmune-induced AP to avoid potential confounders. Besides, few patients admitted to our hospital were diagnosed with these two less common etiologies. Future studies with larger sample sizes are required to confirm our results and to assess the genetic predisposition in other etiologies of AP.

## Conclusions

In conclusion, this study provided preliminary evidence that variant rs651821 is associated with HLAP. Our data revealed the frequency of the risk allele of *APOA5* rs651821 was significantly higher in the HLAP group, and carriers of the CC genotype had increased TG/APOB and FPG levels. Our results suggested that rs651821 might be an important genetic factor for HLAP in southeastern China.
